# *N*-(4-Butanoyl-3-hy­droxy­phen­yl)butanamide

**DOI:** 10.1107/S1600536811001279

**Published:** 2011-01-22

**Authors:** Ming-Ming Yang, Fang-Shi Li, Qi-Sheng Lu, Hao-Wei Wang, Qing Xie

**Affiliations:** a Department of Applied Chemistry, College of Science, Nanjing University of Technology, No. 5 Xinmofan Road, Nanjing 210009, People’s Republic of China

## Abstract

The title compound, C_14_H_19_NO_3_, was prepared *via* the intra­molecular rearrangement of 3-(butanoyl­amino)­phenyl butano­ate in the presence of anhydrous aluminium chloride. The near coplanarity of the aromatic ring, the amide group and the carbonyl group of the butanoyl fragment [N—C—C—C = −179.65 (17) and O—C—C—C = −178.34 (17)°] results from the intra­molecular O—H⋯O and C—H⋯O hydrogen bonds. In the crystal, the mol­ecules form a one-dimensional polymeric structure *via* N—H⋯O inter­actions between their amide groups.

## Related literature

For the synthesis, see: Wang *et al.* (2006 ▶).
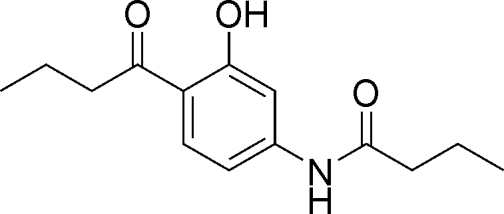


## Experimental

### 

#### Crystal data

C_14_H_19_NO_3_
*M* *_r_* = 249.30
Monoclinic, 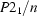

*a* = 6.2870 (13) Å
*b* = 10.008 (2) Å
*c* = 21.680 (4) Å
β = 97.96 (3)°
*V* = 1351.0 (5) Å^3^
*Z* = 4
Mo *K*α radiation
μ = 0.09 mm^−1^
*T* = 293 K
0.30 × 0.20 × 0.20 mm

#### Data collection

Enraf–Nonius CAD-4 diffractometer
Absorption correction: ψ scan (North *et al.*, 1968 ▶) *T*_min_ = 0.975, *T*_max_ = 0.983
2684 measured reflections
2448 independent reflections
1732 reflections with *I* > 2σ(*I*)
*R*_int_ = 0.035
3 standard reflections every 200 reflections  intensity decay: 1%

#### Refinement

*R*[*F* ^2^ > 2σ(*F* ^2^)] = 0.050
*wR*(*F* ^2^) = 0.160
*S* = 1.00
2448 reflections
164 parameters
H-atom parameters constrained
Δρ_max_ = 0.19 e Å^−3^
Δρ_min_ = −0.18 e Å^−3^

### 

Data collection: *CAD-4 Software* (Enraf–Nonius, 1985 ▶); cell refinement: *CAD-4 Software*; data reduction: *XCAD4* (Harms & Wocadlo, 1995 ▶); program(s) used to solve structure: *SHELXS97* (Sheldrick, 2008 ▶); program(s) used to refine structure: *SHELXL97* (Sheldrick, 2008 ▶); molecular graphics: *SHELXTL* (Sheldrick, 2008 ▶); software used to prepare material for publication: *SHELXTL*.

## Supplementary Material

Crystal structure: contains datablocks I, global. DOI: 10.1107/S1600536811001279/gk2334sup1.cif

Structure factors: contains datablocks I. DOI: 10.1107/S1600536811001279/gk2334Isup2.hkl

Additional supplementary materials:  crystallographic information; 3D view; checkCIF report

## Figures and Tables

**Table 1:** Hydrogen-bond geometry (Å, °)

*D*—H⋯*A*	*D*—H	H⋯*A*	*D*⋯*A*	*D*—H⋯*A*
N1—H1*N*⋯O1^i^	0.86	2.29	3.109 (2)	160
O2—H2*A*⋯O3	0.82	1.83	2.552 (3)	146
C6—H6*A*⋯O1	0.93	2.27	2.875 (3)	122

Symmetry code: (i) 
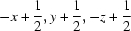
.

**Fig. 1.**
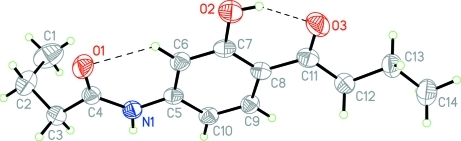
Molecular structure of the title compound with the atom-numbering scheme. Displacement ellipsoids are drawn at the 50% probability level. Hydrogen bonds are shown by dashed lines.

**Fig. 2.**
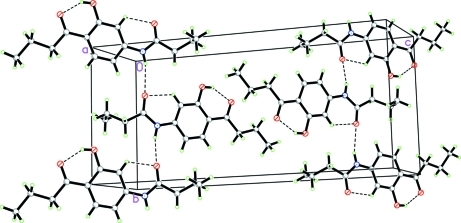
A packing diagram. Hhydrogen bond is shown by dashed lines.

## supplementary crystallographic information

### Comment

The title compound is an important intermediate for the synthesis of an anticoccidial drug Nequinate. It was prepared *via* intramolecular rearrangement of 3-(butanoylamino)phenyl butanoate in 1,2-dichloroethane in the presence of anhydrous aluminium chloride. We report here the crystal structure of the title compound.

The molecular structure is shown in Fig. 1.

In the crystal, molecules are linked *via* intermolecular N—H···O hydrogen bond to form chains.

### Experimental

The title compound (m.p. 381 K) was prepared by a method reported by Wang *et al.* (2006). The crystals were obtained from methanolic solution by slow evaporation.

### Refinement

All H atoms were positioned geometrically, with O—H = 0.82 Å, N—H = 0.86 Å and C—H = 0.93-0.97 Å, and constrained to ride on their parent atoms with *U*_iso_(H) = 1.2U_eq_(C,N,O).

### Crystal data

**Table:**

C_14_H_19_NO_3_	*F*(000) = 536
*M**_r_* = 249.30	*D*_x_ = 1.226 Mg m^−^^3^
Monoclinic, *P*2_1_/*n*	Melting point: 381 K
Hall symbol: -P 2yn	Mo *K*α radiation, λ = 0.71073 Å
*a* = 6.2870 (13) Å	Cell parameters from 25 reflections
*b* = 10.008 (2) Å	θ = 10–13°
*c* = 21.680 (4) Å	µ = 0.09 mm^−^^1^
β = 97.96 (3)°	*T* = 293 K
*V* = 1351.0 (5) Å^3^	Plate, colorless
*Z* = 4	0.30 × 0.20 × 0.20 mm

### Data collection

**Table:**

Enraf–Nonius CAD-4 diffractometer	1732 reflections with *I* > 2σ(*I*)
Radiation source: fine-focus sealed tube	*R*_int_ = 0.035
graphite	θ_max_ = 25.3°, θ_min_ = 1.9°
ω/2θ scans	*h* = 0→7
Absorption correction: ψ scan (North *et al.*, 1968)	*k* = 0→12
*T*_min_ = 0.975, *T*_max_ = 0.983	*l* = −26→25
2684 measured reflections	3 standard reflections every 200 reflections
2448 independent reflections	intensity decay: 1%

### Refinement

**Table:**

Refinement on *F*^2^	Secondary atom site location: difference Fourier map
Least-squares matrix: full	Hydrogen site location: inferred from neighbouring sites
*R*[*F*^2^ > 2σ(*F*^2^)] = 0.050	H-atom parameters constrained
*wR*(*F*^2^) = 0.160	*w* = 1/[σ^2^(*F*_o_^2^) + (0.1*P*)^2^ + 0.080*P*] where *P* = (*F*_o_^2^ + 2*F*_c_^2^)/3
*S* = 1.00	(Δ/σ)_max_ < 0.001
2448 reflections	Δρ_max_ = 0.19 e Å^−^^3^
164 parameters	Δρ_min_ = −0.18 e Å^−^^3^
0 restraints	Extinction correction: *SHELXL97* (Sheldrick, 2008), Fc^*^=kFc[1+0.001xFc^2^λ^3^/sin(2θ)]^-1/4^
Primary atom site location: structure-invariant direct methods	Extinction coefficient: 0.045 (6)

### Special details

**Table:**

Geometry. All e.s.d.'s (except the e.s.d. in the dihedral angle between two l.s. planes) are estimated using the full covariance matrix. The cell e.s.d.'s are taken into account individually in the estimation of e.s.d.'s in distances, angles and torsion angles; correlations between e.s.d.'s in cell parameters are only used when they are defined by crystal symmetry. An approximate (isotropic) treatment of cell e.s.d.'s is used for estimating e.s.d.'s involving l.s. planes.
Refinement. Refinement of *F*^2^ against ALL reflections. The weighted *R*-factor *wR* and goodness of fit *S* are based on *F*^2^, conventional *R*-factors *R* are based on *F*, with *F* set to zero for negative *F*^2^. The threshold expression of *F*^2^ > σ(*F*^2^) is used only for calculating *R*-factors(gt) *etc*. and is not relevant to the choice of reflections for refinement. *R*-factors based on *F*^2^ are statistically about twice as large as those based on *F*, and *R*- factors based on ALL data will be even larger.

### Fractional atomic coordinates and isotropic or equivalent isotropic displacement parameters (Å^2^)

**Table:**

	*x*	*y*	*z*	*U*_iso_*/*U*_eq_	
N1	0.1152 (3)	0.62012 (16)	0.25206 (8)	0.0443 (5)	
H1N	0.1438	0.7034	0.2480	0.053*	
O1	0.2269 (3)	0.41334 (15)	0.22806 (8)	0.0653 (5)	
O2	−0.3406 (3)	0.31968 (14)	0.34457 (8)	0.0624 (5)	
H2A	−0.4387	0.3191	0.3659	0.094*	
O3	−0.6164 (3)	0.41920 (16)	0.40749 (9)	0.0686 (5)	
C1	0.1729 (6)	0.5577 (3)	0.07966 (14)	0.0959 (10)	
H1A	0.1779	0.5148	0.0403	0.144*	
H1B	0.1475	0.6515	0.0732	0.144*	
H1C	0.0590	0.5196	0.0992	0.144*	
C2	0.3834 (5)	0.5374 (2)	0.12083 (11)	0.0668 (7)	
H2B	0.4978	0.5753	0.1006	0.080*	
H2C	0.4104	0.4423	0.1259	0.080*	
C3	0.3882 (4)	0.6008 (2)	0.18464 (11)	0.0547 (6)	
H3A	0.5332	0.5959	0.2067	0.066*	
H3B	0.3502	0.6945	0.1795	0.066*	
C4	0.2373 (3)	0.5346 (2)	0.22330 (10)	0.0445 (5)	
C5	−0.0507 (3)	0.59311 (17)	0.28740 (9)	0.0398 (5)	
C6	−0.1165 (3)	0.46492 (18)	0.29964 (10)	0.0442 (5)	
H6A	−0.0497	0.3914	0.2844	0.053*	
C7	−0.2832 (3)	0.44685 (19)	0.33483 (9)	0.0444 (5)	
C8	−0.3858 (3)	0.55602 (19)	0.35889 (9)	0.0425 (5)	
C9	−0.3162 (3)	0.6842 (2)	0.34437 (9)	0.0453 (5)	
H9A	−0.3830	0.7585	0.3589	0.054*	
C10	−0.1537 (3)	0.70308 (19)	0.30956 (9)	0.0442 (5)	
H10A	−0.1116	0.7892	0.3006	0.053*	
C11	−0.5560 (3)	0.5341 (2)	0.39762 (10)	0.0490 (5)	
C12	−0.6540 (3)	0.6497 (2)	0.42757 (10)	0.0534 (6)	
H12A	−0.7122	0.7119	0.3953	0.064*	
H12B	−0.5417	0.6957	0.4547	0.064*	
C13	−0.8299 (4)	0.6116 (3)	0.46512 (10)	0.0580 (6)	
H13A	−0.7728	0.5484	0.4971	0.070*	
H13B	−0.9440	0.5673	0.4380	0.070*	
C14	−0.9232 (5)	0.7300 (3)	0.49570 (12)	0.0794 (8)	
H14A	−1.0325	0.6996	0.5192	0.119*	
H14B	−0.9846	0.7916	0.4642	0.119*	
H14C	−0.8115	0.7738	0.5231	0.119*	

### Atomic displacement parameters (Å^2^)

**Table:**

	*U*^11^	*U*^22^	*U*^33^	*U*^12^	*U*^13^	*U*^23^
N1	0.0468 (10)	0.0330 (9)	0.0537 (10)	−0.0018 (7)	0.0090 (8)	0.0002 (7)
O1	0.0714 (11)	0.0388 (9)	0.0915 (13)	0.0046 (7)	0.0315 (10)	−0.0037 (8)
O2	0.0734 (11)	0.0376 (9)	0.0804 (11)	−0.0086 (7)	0.0251 (9)	0.0033 (7)
O3	0.0702 (11)	0.0529 (10)	0.0876 (12)	−0.0110 (8)	0.0286 (10)	0.0042 (8)
C1	0.125 (3)	0.087 (2)	0.0665 (18)	0.0120 (19)	−0.0203 (18)	−0.0066 (15)
C2	0.0887 (18)	0.0555 (14)	0.0596 (15)	0.0114 (13)	0.0224 (14)	0.0024 (12)
C3	0.0519 (13)	0.0520 (13)	0.0619 (14)	−0.0067 (10)	0.0140 (11)	−0.0059 (11)
C4	0.0425 (11)	0.0398 (12)	0.0503 (12)	0.0007 (9)	0.0038 (9)	−0.0037 (9)
C5	0.0397 (11)	0.0354 (10)	0.0423 (11)	−0.0005 (8)	−0.0019 (9)	0.0007 (8)
C6	0.0471 (11)	0.0334 (11)	0.0517 (12)	0.0029 (9)	0.0053 (10)	−0.0021 (9)
C7	0.0499 (12)	0.0332 (10)	0.0482 (12)	−0.0042 (9)	−0.0006 (9)	0.0016 (9)
C8	0.0412 (11)	0.0404 (11)	0.0439 (11)	−0.0033 (9)	−0.0004 (9)	0.0013 (9)
C9	0.0477 (12)	0.0390 (11)	0.0496 (12)	0.0025 (9)	0.0073 (10)	−0.0038 (9)
C10	0.0498 (12)	0.0309 (10)	0.0520 (12)	−0.0022 (9)	0.0070 (10)	0.0006 (9)
C11	0.0460 (12)	0.0486 (13)	0.0505 (12)	−0.0040 (10)	−0.0008 (10)	0.0028 (9)
C12	0.0493 (12)	0.0573 (14)	0.0540 (13)	−0.0039 (10)	0.0087 (10)	−0.0011 (11)
C13	0.0551 (13)	0.0691 (15)	0.0512 (13)	−0.0006 (11)	0.0119 (11)	0.0037 (11)
C14	0.089 (2)	0.084 (2)	0.0723 (17)	−0.0043 (15)	0.0356 (16)	−0.0064 (14)

### Geometric parameters (Å, °)

**Table:**

N1—C4	1.357 (3)	C6—C7	1.390 (3)
N1—C5	1.403 (2)	C6—H6A	0.9300
N1—H1N	0.8600	C7—C8	1.406 (3)
O1—C4	1.221 (2)	C8—C9	1.405 (3)
O2—C7	1.348 (2)	C8—C11	1.466 (3)
O2—H2A	0.8200	C9—C10	1.365 (3)
O3—C11	1.239 (3)	C9—H9A	0.9300
C1—C2	1.504 (4)	C10—H10A	0.9300
C1—H1A	0.9600	C11—C12	1.500 (3)
C1—H1B	0.9600	C12—C13	1.510 (3)
C1—H1C	0.9600	C12—H12A	0.9700
C2—C3	1.519 (3)	C12—H12B	0.9700
C2—H2B	0.9700	C13—C14	1.515 (3)
C2—H2C	0.9700	C13—H13A	0.9700
C3—C4	1.504 (3)	C13—H13B	0.9700
C3—H3A	0.9700	C14—H14A	0.9600
C3—H3B	0.9700	C14—H14B	0.9600
C5—C6	1.385 (2)	C14—H14C	0.9600
C5—C10	1.396 (3)		
			
C4—N1—C5	129.76 (17)	O2—C7—C8	121.95 (19)
C4—N1—H1N	115.1	C6—C7—C8	121.46 (18)
C5—N1—H1N	115.1	C9—C8—C7	116.97 (18)
C7—O2—H2A	109.5	C9—C8—C11	122.65 (18)
C2—C1—H1A	109.5	C7—C8—C11	120.38 (18)
C2—C1—H1B	109.5	C10—C9—C8	122.01 (18)
H1A—C1—H1B	109.5	C10—C9—H9A	119.0
C2—C1—H1C	109.5	C8—C9—H9A	119.0
H1A—C1—H1C	109.5	C9—C10—C5	120.00 (18)
H1B—C1—H1C	109.5	C9—C10—H10A	120.0
C1—C2—C3	112.9 (2)	C5—C10—H10A	120.0
C1—C2—H2B	109.0	O3—C11—C8	120.2 (2)
C3—C2—H2B	109.0	O3—C11—C12	119.15 (19)
C1—C2—H2C	109.0	C8—C11—C12	120.61 (18)
C3—C2—H2C	109.0	C11—C12—C13	114.48 (19)
H2B—C2—H2C	107.8	C11—C12—H12A	108.6
C4—C3—C2	112.88 (19)	C13—C12—H12A	108.6
C4—C3—H3A	109.0	C11—C12—H12B	108.6
C2—C3—H3A	109.0	C13—C12—H12B	108.6
C4—C3—H3B	109.0	H12A—C12—H12B	107.6
C2—C3—H3B	109.0	C12—C13—C14	113.3 (2)
H3A—C3—H3B	107.8	C12—C13—H13A	108.9
O1—C4—N1	123.21 (19)	C14—C13—H13A	108.9
O1—C4—C3	122.04 (19)	C12—C13—H13B	108.9
N1—C4—C3	114.76 (18)	C14—C13—H13B	108.9
C6—C5—C10	119.95 (18)	H13A—C13—H13B	107.7
C6—C5—N1	123.20 (17)	C13—C14—H14A	109.5
C10—C5—N1	116.84 (16)	C13—C14—H14B	109.5
C5—C6—C7	119.58 (18)	H14A—C14—H14B	109.5
C5—C6—H6A	120.2	C13—C14—H14C	109.5
C7—C6—H6A	120.2	H14A—C14—H14C	109.5
O2—C7—C6	116.59 (17)	H14B—C14—H14C	109.5
			
C1—C2—C3—C4	67.0 (3)	C6—C7—C8—C11	−177.91 (18)
C5—N1—C4—O1	−5.1 (3)	C7—C8—C9—C10	−1.3 (3)
C5—N1—C4—C3	174.86 (19)	C11—C8—C9—C10	178.29 (19)
C2—C3—C4—O1	46.3 (3)	C8—C9—C10—C5	−0.2 (3)
C2—C3—C4—N1	−133.7 (2)	C6—C5—C10—C9	1.4 (3)
C4—N1—C5—C6	1.0 (3)	N1—C5—C10—C9	−179.87 (17)
C4—N1—C5—C10	−177.72 (19)	C9—C8—C11—O3	177.71 (19)
C10—C5—C6—C7	−1.0 (3)	C7—C8—C11—O3	−2.7 (3)
N1—C5—C6—C7	−179.65 (17)	C9—C8—C11—C12	−4.4 (3)
C5—C6—C7—O2	179.43 (17)	C7—C8—C11—C12	175.14 (18)
C5—C6—C7—C8	−0.6 (3)	O3—C11—C12—C13	−3.1 (3)
O2—C7—C8—C9	−178.34 (17)	C8—C11—C12—C13	179.01 (18)
C6—C7—C8—C9	1.7 (3)	C11—C12—C13—C14	179.02 (19)
O2—C7—C8—C11	2.1 (3)		

### Hydrogen-bond geometry (Å, °)

**Table:**

*D*—H···*A*	*D*—H	H···*A*	*D*···*A*	*D*—H···*A*
N1—H1N···O1^i^	0.86	2.29	3.109 (2)	160
O2—H2A···O3	0.82	1.83	2.552 (3)	146
C6—H6A···O1	0.93	2.27	2.875 (3)	122

Symmetry codes: (i) −*x*+1/2, *y*+1/2, −*z*+1/2.
